# Tortuous vertebral artery triggering vertebral foramen expansion and radiculopathy in a 19-year-old patient: a case report

**DOI:** 10.1186/s13256-020-02493-8

**Published:** 2020-09-28

**Authors:** Ibrahim F. Naldemir, Elif Nisa Unlu, Omer Onbas

**Affiliations:** Department of Radiology, Duzce University, Konuralp Yerleşkesi Konuralp Merkez, 81620 Düzce, Turkey

**Keywords:** Vertebral artery loop formation, Tortuous vertebral artery, Widening of vertebral foramen, VALF

## Abstract

**Background:**

Arterial tortuosity is a widespread condition commonly occurring in cerebral arteries and often associated with the elderly. Moderate arterial tortuosity is often not symptomatic, but if there is severe tortuosity, various symptoms may occur, depending on the localization. In the literature, many factors have been reported as causing tortuosity in the vertebral arteries. In this case report, considering the age of our patient, we propose that, in addition to these previously reported reasons, congenital factors may also contribute to this situation.

**Case presentation:**

We present a case of a 19-year-old Turkish patient with a tortuous vertebral artery causing pain and tingling in the right shoulder and neck. Magnetic resonance imaging revealed vertebral foramen enlargement thought to be secondary to a loop formation in the vertebral artery. The diagnosis was confirmed by magnetic resonance angiography.

**Conclusions:**

There are many causes of cervical radiculopathy. Arterial tortuosity, a rare cause of radiculopathy, should be considered as a differential diagnosis. Consideration of the existence of this variation is of great importance in preventing possible dangerous complications during surgery.

## Introduction

Arterial tortuosity is not an uncommon condition and is especially common in cerebral arteries. Moderate arterial tortuosity is often not symptomatic; however, depending on localization and severity, tortuosity may lead to various symptoms. Tortuous vertebral arteries are often associated with the elderly. Here, we present a case of a young patient with a tortuous vertebral artery enlarging the vertebral foramen, extending the adjacent neural foramen, and causing intermittent radiculopathy.

## Case presentation

**Fig. 1:**
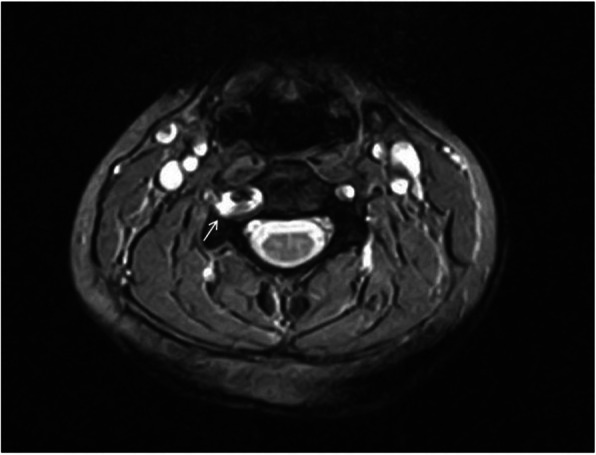
Axial T2AG, enlargement of the right vertebral foramen, thinning of the peduncle and erosion of the lateral part of the vertebral corpus (white arrow)

**Fig. 2:**
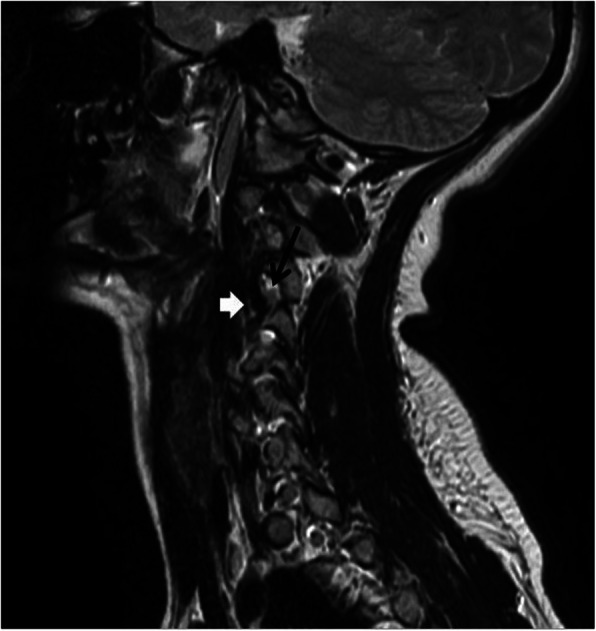
Sagittal T2AG, vertebral artery at the C3-4 level extending to the right neural foramen and contacting the nerve root (thick arrow). Nerve root (black arrow)

**Fig. 3:**
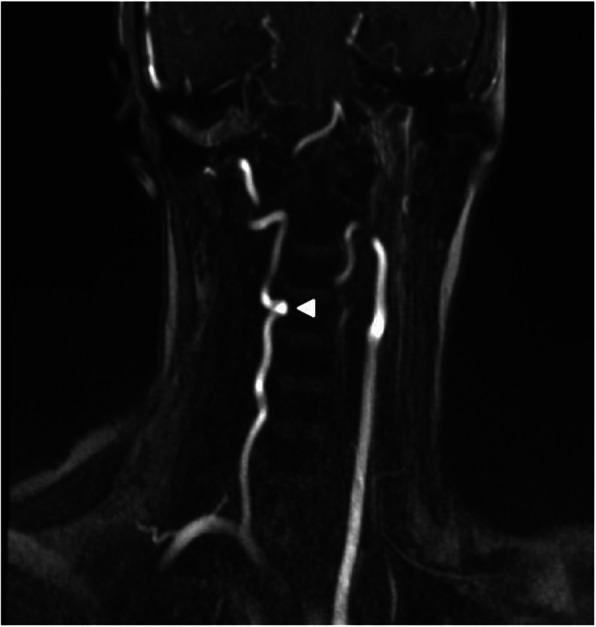
MR angiography, tortuous vertebral artery at the level of C4 vertebra (arrowhead)

A 19-year-old Turkish woman with no history of trauma was admitted to the hospital due to increased right shoulder and neck pain at 2-month intervals. Physical examination revealed cervical axis flattening, right trapezius and paravertebral muscle spasm, tingling in the area, and limitation of neck movement. Cervical magnetic resonance (MR) revealed enlargement of the right vertebral foramen at the C3–C4 level, scalloping on the right side of the vertebral corpus, thinning on the right pedicle, and compression of the nerve root by the vascular structure on the right side (Figs. 1 and 2). Neither bone pathology nor intervertebral disc herniation was observed. The described findings were thought to be due to pathology present in the vertebral artery. Vertebral artery loop formation (VALF) was observed at the C4 vertebral level upon examination using contrast-enhanced magnetic resonance angiography (MRA) (Fig. 3). Considering the age of the patient, computed tomography (CT) was not performed to prevent exposure to ionizing radiation. In addition, because the diagnosis was not in doubt, it was thought that CT would not provide any additional clinical contribution. The patient was referred to the neurosurgery clinic. Considering the surgical risks, it was decided to follow the patient with conservative methods, and nonsteroidal anti-inflammatory drugs were recommended for pain control if necessary.

## Discussion

Cervical radiculopathy may occur frequently due to disc disease. Also, neoplasms, cystic lesions, metabolic and vascular diseases, and congenital conditions should be considered when researching differential diagnoses [1]. VALF is one of the rare conditions that can cause radiculopathy [2]. This entity is often asymptomatic, but it can be symptomatic if it compresses a nerve root or the spinal cord. Widening of the cervical neural foramen is a very rare entity of VALF. Although this rare condition is described in the literature in the elderly [2–4], it is even rarer in young patients such as ours. Although the etiology of VALF is not clear, hypotheses have been reported in the literature, including as a development secondary to cervical spondylotic degenerative changes, or due to atherosclerotic disease, or due to vertebral artery elongation caused by narrowing of disc space or trauma [5–7]. However, because our patient was young, had no history of trauma, and lacked possible etiologic pathologies described in the literature, we assumed congenital causes may also contribute to the etiology of VALF. Ono *et al.* reported that VALF is most commonly seen at the C4–C5 level, followed by C3–C4, and most frequently occurs on the left side [1]. In cervical radiographs, erosion of the adjacent bone structure due to arterial pulsation as well as vertebral foramen and neural foramen expansion can be seen [2]. Contrast-enhanced CT can provide a clear determination of VALF and its level, but MRA is more appropriate to prevent the patient from being exposed to ionizing radiation. For patients with cervical MR examinations, if there is an expansion of the vertebral foramen, VALF should be suspected and MRA should be performed [3]. It has been reported in the literature that conservative methods rarely succeed in treatment of this condition [8]. In addition, surgical approaches such as microvascular decompression and foraminotomy may be treatment options [2]. However, it is important to consider serious risks that may affect the quality of life, especially in young patients, such as nerve root damage or vertebrobasilar system bleeding. Therefore, conservative management modalities are preferred in patients whose symptoms do not affect their daily lives. The treatment method should be determined on the basis of the patient’s age, symptoms, living conditions, and location of the vascular loop. A differential diagnosis of VALF and exclusion of other pathologies may be difficult with noncontrast examinations; however, with contrast-enhanced CT-MR or MRA, a VALF diagnosis can be easily confirmed [3].

## Conclusion

Arterial tortuosity is a common condition in the elderly and may be symptomatic or not, depending on its localization. In young people, tortuosity in vertebral arteries is rare and may rarely be symptomatic. Consideration of this entity in the differential diagnosis of vertebral foramen enlargement and radiculopathy ensures the prevention of major complications in patients who may undergo surgery.

## Data Availability

Not applicable.
